# How I do it: combined interposition-transposition technique for microvascular decompression in primary hemifacial spasm

**DOI:** 10.1007/s00701-025-06472-0

**Published:** 2025-03-18

**Authors:** Francesco Tengattini, Fabio Calbucci, Ignazio Borghesi, Riccardo Draghi

**Affiliations:** 1 Department of Medical and Surgical Specialties, Division of Neurosurgery, Radiological Sciences and Public Health, University of Brescia, Brescia, Italy; 2 Department of Neurosurgery, Maria Cecilia Hospital, GVM Care&Research, Cotignola, Italy

**Keywords:** Hemifacial spasm, Step-by-step surgery, 2-Dimensional operative video, Microvascular decompression, Neuromonitoring, Neurovascular conflict

## Abstract

**Background:**

Two surgical microvascular decompression techniques to treat primary hemifacial spasm (HFS) are described in literature. The “interposition” uses different materials as “spacers” while the “transposition” consists in suturing the offending vessel to the petrous bone dura mater. In the literature is described a transposition surgery adopting self-adhesive materials reporting promising results.

**Method:**

We describe a combination of these two techniques using autologous muscle and Tachosil^®^. The step-by-step surgical principles are described in the article and clarified in a video.

**Conclusion:**

This procedure is effective to cure the HFS especially in case of large arteries when interposition only is insufficient.

**Supplementary Information:**

The online version contains supplementary material available at 10.1007/s00701-025-06472-0.

## Introduction and relevant surgical anatomy

The primary hemifacial spasm (pHFS) is mainly caused by neurovascular conflict (NVC) at the VII cranial nerve’s root exit zone (REZ) [2]. The definitive treatment is the microvascular decompression (MVD) that can be achieved by two different techniques as described in literature. The “interposition” one is the most common, using several materials such as autologous muscle, absorbable gelatins, Ivalon, Teflon or Dacron. It is simple and safe but may have some drawbacks, as neo-compression, mobilization of the interposed material and arachnoiditis [6]. The “transposition” is similarly effective and usually performed with «sling» technique stitching the tentorial or the petrous dura mater. While the latter technique has the advantage that no material is left in contact with the nerve, on the other hand this is a technically demanding surgery with non-negligible risk of surrounding structures injuries [3].

In the recent years, some authors proposed a simpler transposition technique with the adoption of self-adhesive materials such as Tachosil^®^ and TachoComb^®^ with promising results. It has the advantage to be less technically demanding, reducing potential risks to CPA neurovascular structures [5, 1, 4].

Especially in case of large offending vessels, we adopt an “hybrid” technique, since it takes advantage of a classical interposition technique using muscle fragments and the “Double-Stick Tape” transposition described by Ichikawa et al. [1]. The interposition step let the Vertebral artery (VA) to get closer to the posterior petrous bone, otherwise too far to allow a direct bonding of the vessel. Moreover, the muscle pad ensures a protection of the facial nerve exit zone.

Although this technique is easier to perform then the classical «sling» technique, the mobilization of the CPA arteries must be always watchful and gentle, avoiding any vessel kink or damage to its perforating branches.

## Description of the technique

### Clinical presentation

**Fig. 1:**
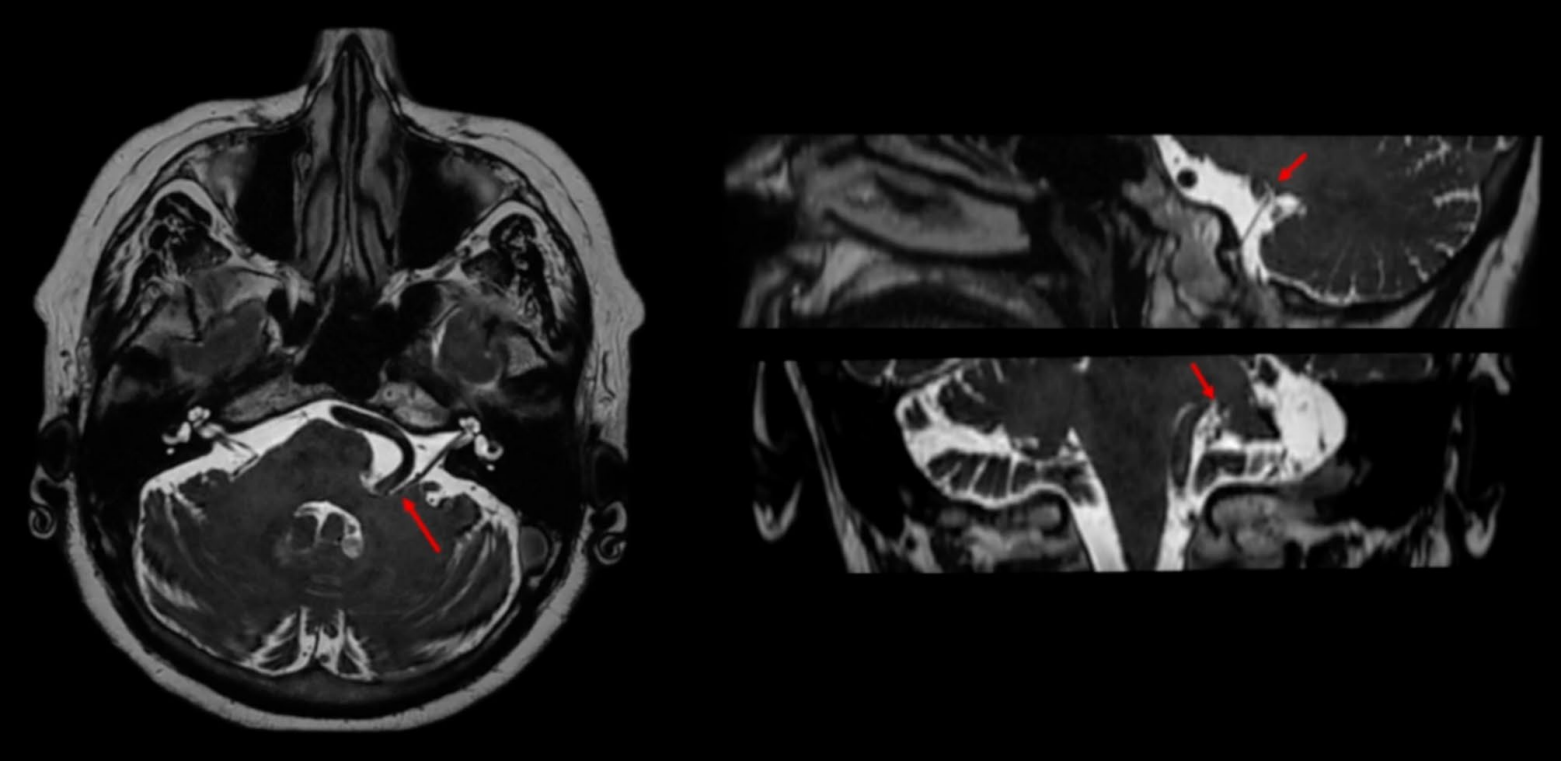
The brain MRI with FIESTA sequences in three planes showed a potential neurovascular conflict between the left vertebral artery and the facial nerve along its proximal tract near the root exit zone pointed in with the red arrow. The vertebral artery was aberrant in his course with a loop occupying the left cerebellopontine angle

The basic steps of the surgery and the surgical considerations are described using the illustrative case of a 63-year-old woman. The patient started complaining involuntary contraction twiches of the left orbicularis oculi and performed a Brain MRI showed a potential neurovascular conflict between the left vertebral artery and the facial nerve, at the level of the pontomedullary junction (Fig. 1). The involuntary contraction twitches of the left face appeared progressively worsening in extension and frequency, they extended from orbicularis oculi muscle to the platysma without facial paresis configuring a typical primary Hemifacial Spasm. After evaluation of the clinical and neuroradiological features, microvascular decompression of the left VII nerve was proposed.

### Surgical steps

**Fig. 2:**
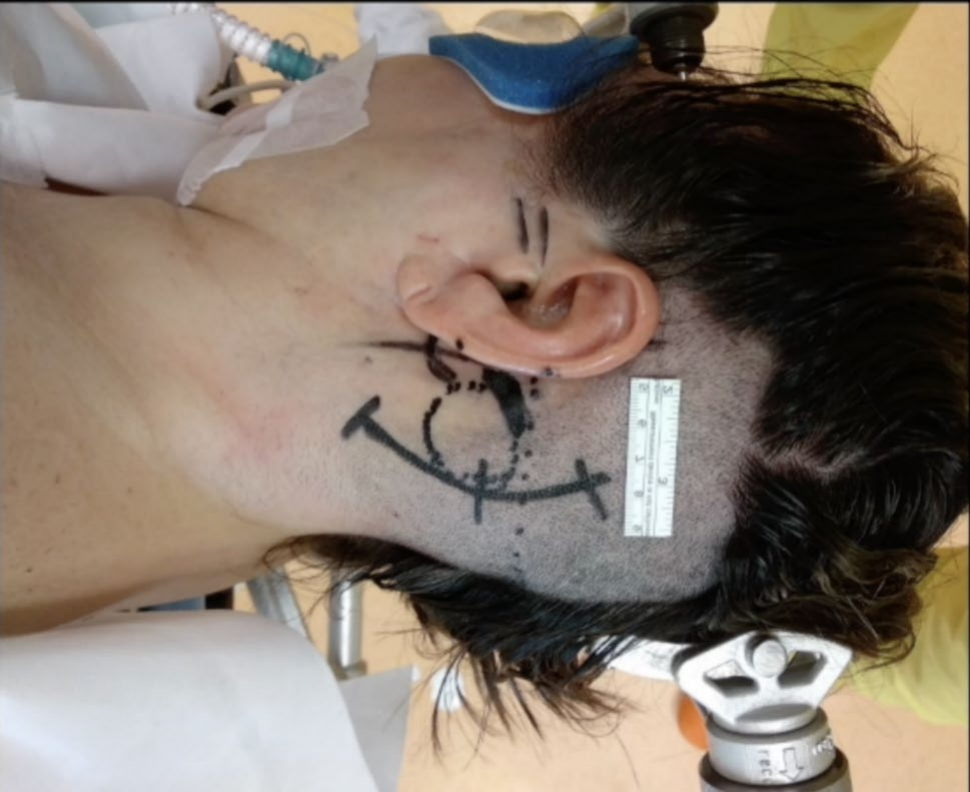
The patient is positioned Park Bench on the right side, the head is flexed and fixed with Mayfield clamp. A left Curvilinear retromastoid skin incision was performed, 3 cm behind the ear

**Fig. 3:**
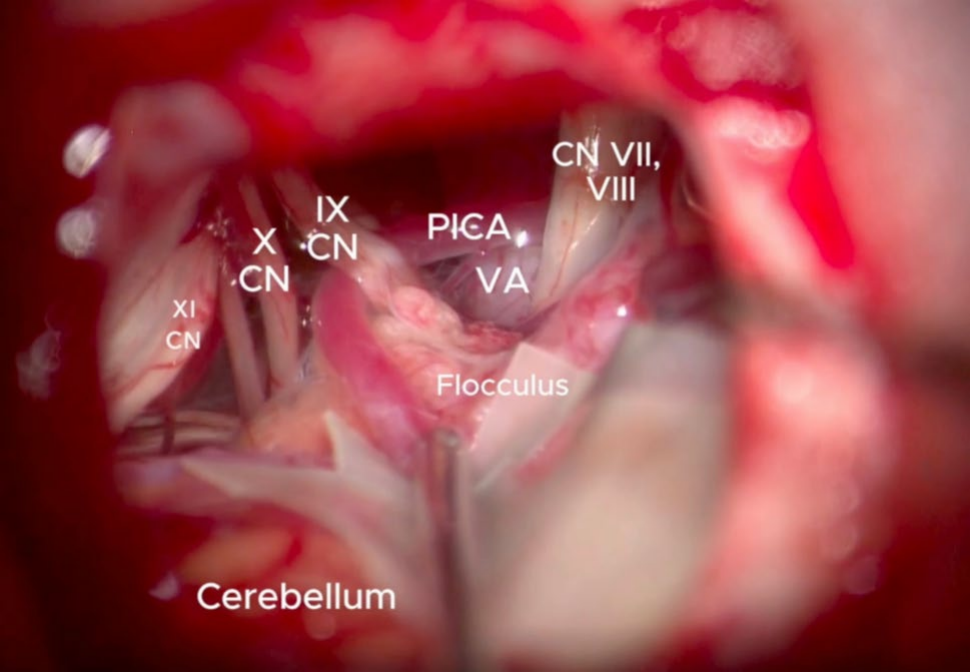
View of the vascular and nervous structures in the ponto-cerebellar angle after an accurate arachnoid membrane dissection

**Fig. 4:**
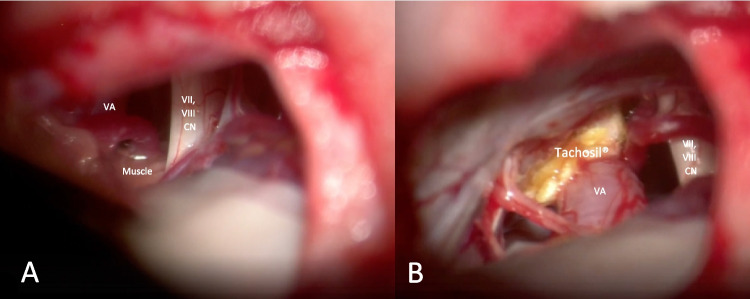
Description of the hybrid technique. In the interposition step (**A**) the autologous muscle fragment is positioned between the vertebral artery and the acoustic facial bundle and in the transposition step (**B**) the Tachosil^®^ is used to attach the VA to the petrous bone

The patient positioning was Park Bench with the right side down. The head was gently flexed and fixed with Mayfield clamp. A left Curvilinear retromastoid skin incision was performed and a retrosigmoid craniectomy was made. The skin incision was curvilinear 3 cm behind the ear (Fig. 2). The lateral occipital squama and temporal retromastoid bone were exposed and an infratentorial craniotomy or craniectomy at the transverse sinus—sigmoid sinus angle was made. A C-shaped dura opening was performed and the flap was fold over the mastoid. The Ponto cerebellar cistern was gradually opened, allowing slow and progressive CSF drainage creating space without the need of excessive retraction. The arachnoid membranes in the ponto-cerebellar angle were gently dissected the acoustic-facial boudle and the IX nerve were reached, allowing an infrafloccular approach to the pontomedullary sulcus (Fig. 3). A careful cerebellar retraction permitted to identify the VII and VIII nerves root exit zones and the neurovascular conflict. The vessels involved were the Vertebral Artery and the Anterior inferior cerebellar artery which were progressively mobilised away from the root exit zone of the facial nerve. An effective removal of the vertebral artery from the VII nerve root exit zone was achieved by the interposition of muscle fragments, allowing an approach of the vessel to the posterior petrous bone. The “new anatomy” of the VA and its branches was explored, in order to detect any possible kink. The not-adhesive side of the Tachosil^®^ fleece is coated by the fibrin glue and the “double-stick tape” [1] is positioned between the VA and the dura mater of the petrous bone. The VA is then gently compressed until the glue gets hard. After checking the final decompression result and haemostasis, dura closure, bone defect covering, and plane closure was performed (Fig. 4).

### Indications

A typical history and clinical picture of typical left hemifacial spasm that resulted to be persistent and psychologically disabling represented the rationale of the procedure. The Brain MRI, especially with CISS or FIESTA sequences, excluding a secondary hemifacial left spasm and documenting a close proximity between VA and the acoustic facial bundle at the pontomedullary sulcus should be performed. However, if patient’s clinical history and examination are typical for hemifacial spasm, detection on MRI of possible neurovascular conflict is not necessary to perform microvascular decompression.

**Fig. 5:**
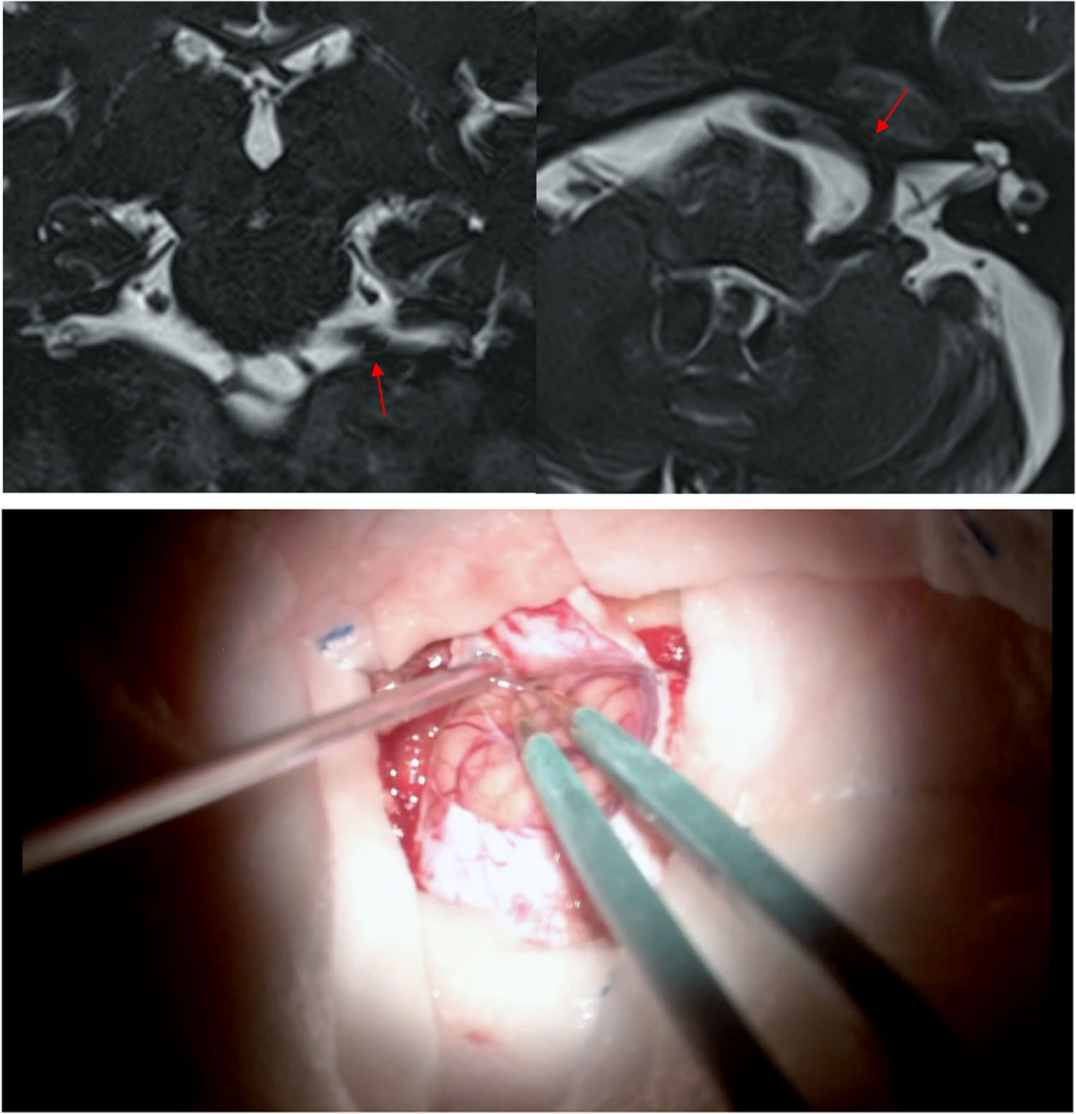
The brain MRI with FIESTA sequences in the coronal and axial planes showed the fibrous attachment between the left VA and the petrous bone dura mater (red arrow)

The “hybrid” technique we have described can be specifically used in case of large offending arteries, when simple interposition is considered insufficient to win the pulsatile force and the “shape-memory” of the vessel. In agreement with Ichikawa et al., we believe that Tachosil^®^, if the correct size is used, have enough adhesive strength to keep a large intracranial vessel sticked to the dura of the petrous bone until the permanent fibrous tissue is developed around TachoSil^®^. The two years follow-up MRI (Fig. 5) showed the VA attached to the dura mater by the neo formed fibrous tissue.

### Limitations

Even though an excessive manipulation must be avoided, the surgical cavity is small and rich in vasculo-nervous structures. For this reason, despite careful microsurgical technique, the microvascular decompression using the interposition-transposition technique involves some degree of undue manipulation of the structures of the PCA. The indication for the use the “hybrid” technique in less strong when dealing with small and easily dislocated offending vessels, in that case a simple interposition technique could be adequate.

### Specific perioperative considerations

Real-time Electrophysiological intraoperative monitoring feedback is necessary to minimize the risk of postoperative cranial nerves deficits due to excessive manipulation. In order to create an adequate working space, the Ponto cerebellar cistern has to be gently opened and a gradual CSF drainage must be accomplished. This simple maneuver guarantees a complete control of the structures into the PCA avoiding excessive cerebellar retraction and contusions. Furthermore, an accurate dissection of the arachnoid membranes up to the pontomedullary sulcus and the root exit zones clarify the view and permits an easier manipulation of the vessels (e.g. Vertebral artery).

### Specific information to give to the patient about surgery and potential risks

Preoperatively, patients are informed about the alternative therapies and why there are not chosen. In fact, different non-surgical treatments, such as pharmacological treatment with antidepressants and anticonvulsants and Botulinum toxin therapy should be considered but surgery is the only curative for this pathology. Considering the transitory effect of the other palliative treatments and the severity of the clinical picture, Microvascular Decompression (MVD) appeared to be the best option. Additionally, this procedure is less technically demanding, safer, time-saving and effective in solving the NVC involving the VA then the other interposition and/or transposition techniques.

Finally, the risks of the procedure included bleedings comprehending intracranial hematoma, Infection, CSF leak and cerebellar contusion or infarction as well as facial paresis, brainstem lesion, hearing loss and tinnitus, gait disturbances and cranial nerve damage. It is important that patients are informed that the facial spasm may not be solved after surgery.

## Ten key points

1. Between the two main surgical techniques for the treatment of primary hemifacial spasm the “interposition” one is the most common, simple and safe but may cause neo-compression, mobilization of the interposed material and arachnoiditis. The “transposition” one does not include the placing of material is left in contact with the nerve, but it is technically demanding and risky
2. Pharmacological treatment with antidepressants and anticonvulsants and Botulinum toxin therapy should be considered but surgery is the only curative for this pathology
3. The “hybrid” technique is inspired by both classic techniques. The interposition step let the Vertebral artery to get closer to the posterior petrous bone while the transposition step ensures the artery does not move
4. A history and a clinical picture of typical left hemifacial spasm represent the rationale of the procedure. The neurovascular conflict could not be seen in the brain MRI
5. Real-time Electrophysiological intraoperative monitoring feedback is necessary to minimize complications
6. A retrosigmoid craniectomy with an infrafloccular approach is effective to reach the pontomedullary sulcus.
7. The progressive CSF outflow and a gentle arachnoid membranes dissection are essential to reach the nerve root exit zones avoiding excessive cerebellar retraction
8. This new interposition- transposition technique could be adopted especially in case of large offending vessels when simple interposition is considered insufficient to win the pulsatile force and the “shape-memory” of the vessel.
9. The risks of the procedure include bleedings, Infection, CSF leak and cerebellar contusion or infarction, facial paresis, brainstem lesion, hearing loss and tinnitus, gait disturbances and other cranial nerve damage.
10. In our experience this technique resulted effective in solving the primary hemifacial spasm

## Supplementary Information

Below is the link to the electronic supplementary material.

ESM 1 (MP4 260 MB)

## Data Availability

No datasets were generated or analysed during the current study.
